# Supplementary material for: RNA viruses can exploit self-cleaving ribozymes for translation initiation
Source: Nucleic Acids Res. 2026 Nov 2;54(20):gkag1106. doi: 10.1093/nar/gkag1106 (PMC13725137; doi:10.1093/nar/gkag1106)

Supplementary Figures and Tables

Supplementary Table 1. Accession numbers of all the RNA virus genomes/segments containing self-cleaving ribozymes identified in this study.

EXCEL FILE

Supplementary Table 2. Oligonucleotide sequences and their specific use in this work.

Oligonucleotide Name	Sequence (5' to 3')	Use
BcCV1-RNA1-77Rev	CAAAGATGCGTTTTCCCATGC	RACE
BcCV1-RNA1-87Rev	TGCTTTGCAGCACCGTTTA	RACE
BcCV1-RNA1-88NRev	TCGTCTGTGTAGCAACACTAAC	RACE
BcCV1-RNA2-81Rev	GCGCAAGCAACCAGGTTCA	RACE
BcCV1-RNA2-82Rev	CCTTCCATTGTGCTATTAAGGTAT	RACE
BcCV1-RNA3-85Rev	CTCTTCATGTTGCATGAATTCG	RACE
BcCV1-RNA3-86Rev	TGCAGACGTGTTTTCCATTGT	RACE
Fusari-5000Rev	TGTTTCCTTGACGCTCGTGT	RACE
Fusari-4800For	ACCCGGAGAGCTCAAATGTT	RACE
Fusari-4980Rev	GTAACTTAAGAAGTTTGGCT	RACE
GcCV1_RNA3_1163Rev	AATCTCTCCGCCGCTGTCGT	RACE
GcCV1_RNA3_1104Rev	TGTTCTCCCGTTCCTCGGTG	RACE
HvV145S-2-F_BstEII	ATAGGTCACCGATAAAAACAAAAAATCC	DL
OFluc-5ter-R	GTTCTTGGCGTCCTCCATG	DL
HvV145S-2-d60F_BstEII	ATAGGTCACCATTTAAAAACAATTAATTTGTTTAATTT	DL
HvV145S-2-d120F_BstEII	ATAGGTCACCACTGTCGAGCCGTCTGATG	DL
Del-RZregion-R	TCAATAATCTAGTCTAACTGTGCCAGACAG	DL
Del-RZregion-F	CAGTTAGACTAGATTATTGAGGTTAGTAGAACA	DL
G159T-R	TCCAGCGTTT(A)GTGACCCTGAGGTGCTACT	DL
G159T-F	TCAGGGTCAC(T)AAACGCTGGACGTAACGGC	DL
Del-CUGAU-R	GGTGCTACTCACGGCTCGACAGTCTAACT	DL
Del-CUGAU-F	GTCGAGCCGTGAGTAGCACCTCAGGGTCA	DL
HvV145S-2-5UTR-R_NotI	ATAGCGGCCGCCATCTTGTTTGTTTGACAGTGT	DL

Supplementary Figures

Supplementary Fig. 1. (A) Detailed consensus models and secondary structures of the hammerhead ribozymes either with long helix II (left, based on 26 ribozyme sequences) or short helix II (right, based on 62 ribozyme sequences) from chrysovirus genomes. (B) Consensus model of the hammerhead ribozyme present in most fusarivirus genomes (based on 123 ribozyme sequences). The legend inset applies to the three models shown. (C) Three representative examples of hammerhead ribozymes from fusarivirus genomes with variable stem lengths for helix I, II and III. The site of ribozyme-mediated RNA cleavage is identified by an arrowhead. Consensus models were built as previously described(40).

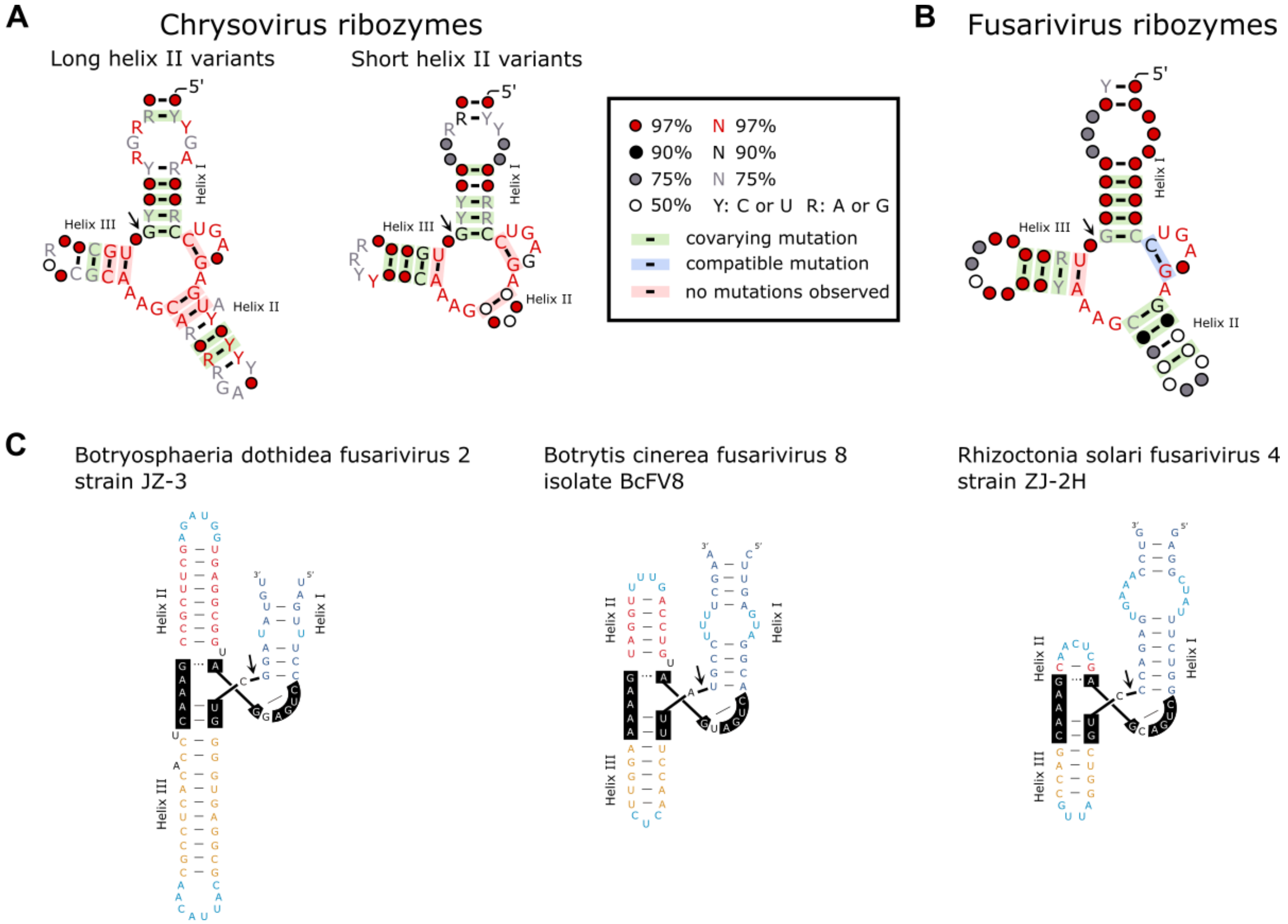

Supplementary Fig. 2. Representative assays of self-cleavage kinetics for four hammerhead ribozymes encoded in fungal and plant RNA viruses. (A) Structure of the hammerhead ribozyme from Verticillium dahliae chrysovirus 1, RNA 1 (left) and a 10% PAGE showing the resulting RNAs after different transcription times (standard conditions, pH 7.5, 1 mM Mg^{2+}) (right). (B) Structure of the hammerhead ribozyme from Brassica campestris chrysovirus (left) and two 10% PAGEs showing post-transcriptional assays of self-cleavage with purified RNA of this ribozyme incubated at pH 8.5 and 1 (center) or 10 mM Mg^{2+} (right). (C) Structure of the hammerhead ribozyme from Penicillium chrysogenum virus, RNA 1 (left) and a 10% PAGE showing a post-transcriptional assay of self-cleavage with purified RNA of this ribozyme incubated at pH 8.5 and 1 or 10 mM Mg^{2+} (right). (D) Structure of the hammerhead ribozyme from the Rosellinia necatrix megabirnavirus 1 (RnMBV1) (left) and a 10% PAGE showing a co-transcriptional self-cleavage assay. Observed constant rates (k_{obs}) obtained from three independent experiments for each ribozyme and experimental condition are indicated.

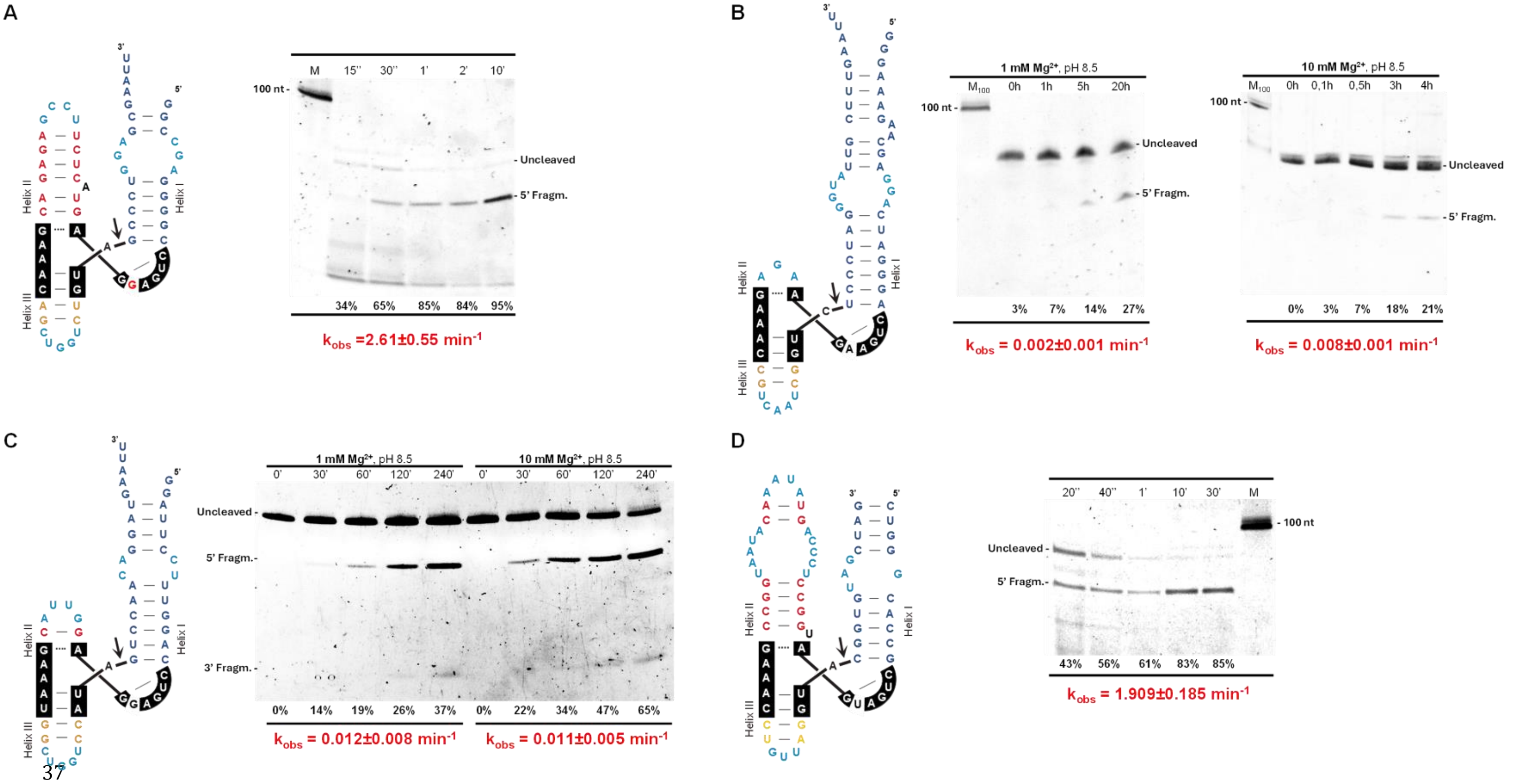

Supplementary Fig. 3. (A) Representative examples of hammerhead ribozymes (HHRs) detected in the UTRs of two ssRNA fusariviruses. (B) An example of a dsRNA megabirnavirus-like genome showing the presence of a HHR in its 5'-UTR and a deltavirus ribozyme encoded within the predicted RNA dependent RNA polymerase (RdRp) ORF. Sites of ribozyme self-cleavage are indicated by arrows.

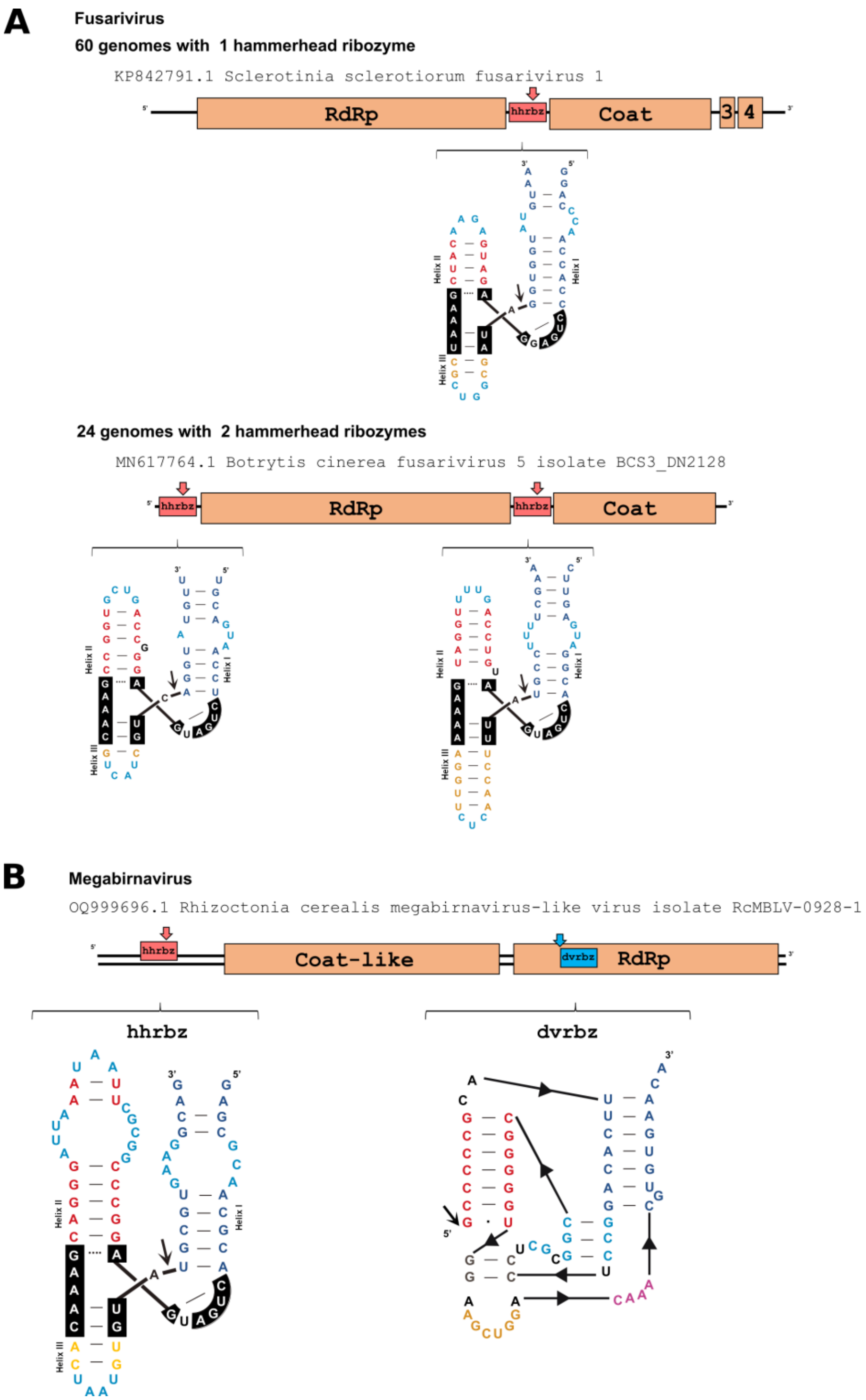

Supplementary Fig. 4. Predicted subgenomic circRNAs detected in (A) Sclerotium rolfsii, (B) Telephora terrestris, (C) Letinula edodes and (D) Phlebiopsis gigantea toti-like mycoviruses containing close ribozyme pairs. Resulting RNAs after double self-cleavage show quasi-rod like structures (circular Jupiter plots are shown at the right), sizes multiple of 3 nt, and absence of stop codons (potentially encoding endless ORFs with protein sequences shown at the insets) as similarly described for zetaviruses (in panel (E), two previously reported Zetavirus genomes are shown for comparison)(31, 34). Hyp; hypothetical. polyprot; polyprotein. HHR; hammerhead ribozyme. DVR; deltavirus ribozyme. RdRp; RNA dependent RNA polymerase.

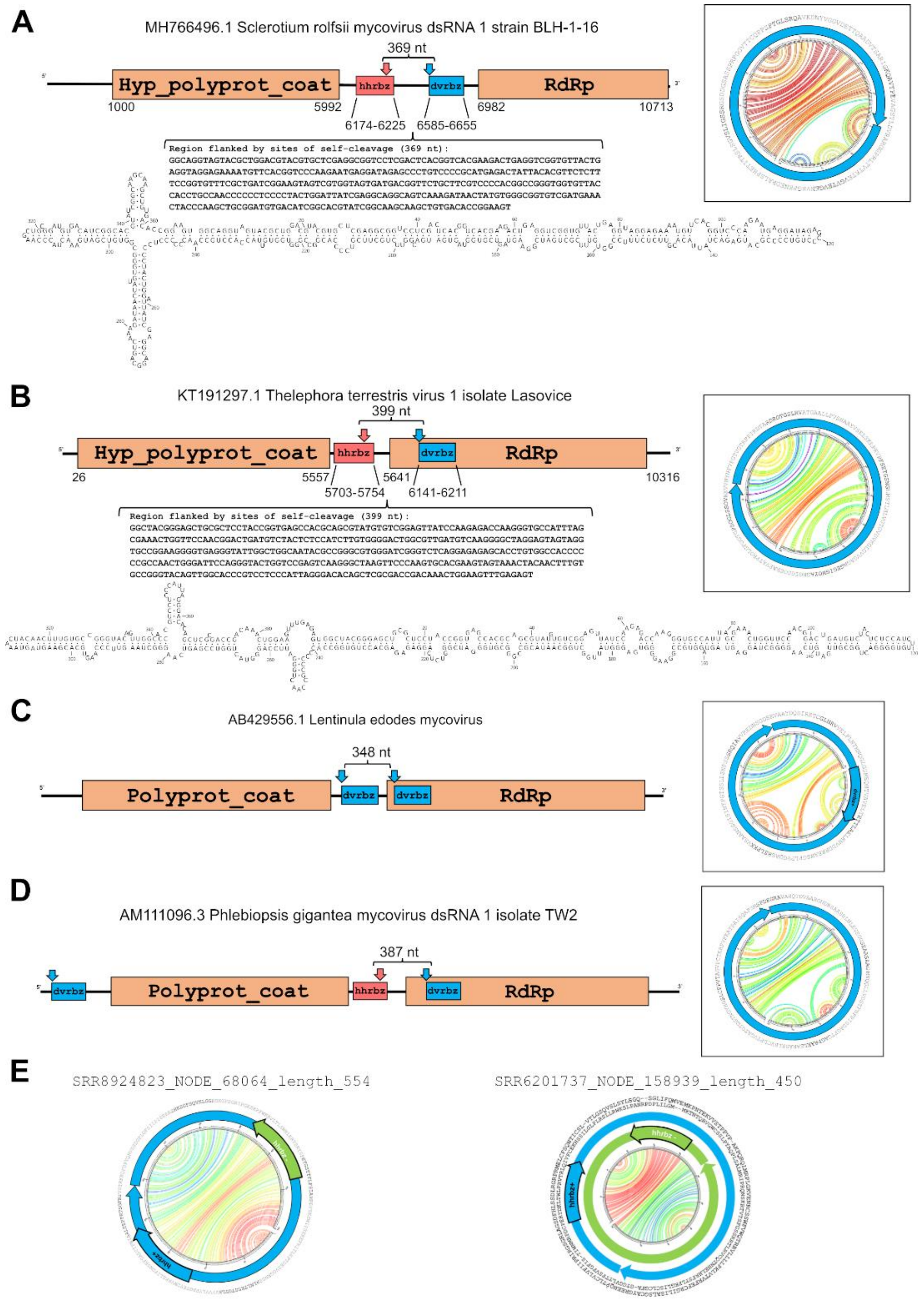

Supplementary Fig. 5. Twister ribozymes (TWR) motifs detected in the RNA genomes of (A) phelgivirus-like and (B) tombusvirus-like sequences. Highly conserved nucleotides and unexpected changes in the consensus structure of most TWR motifs are depicted in red and blue, respectively.

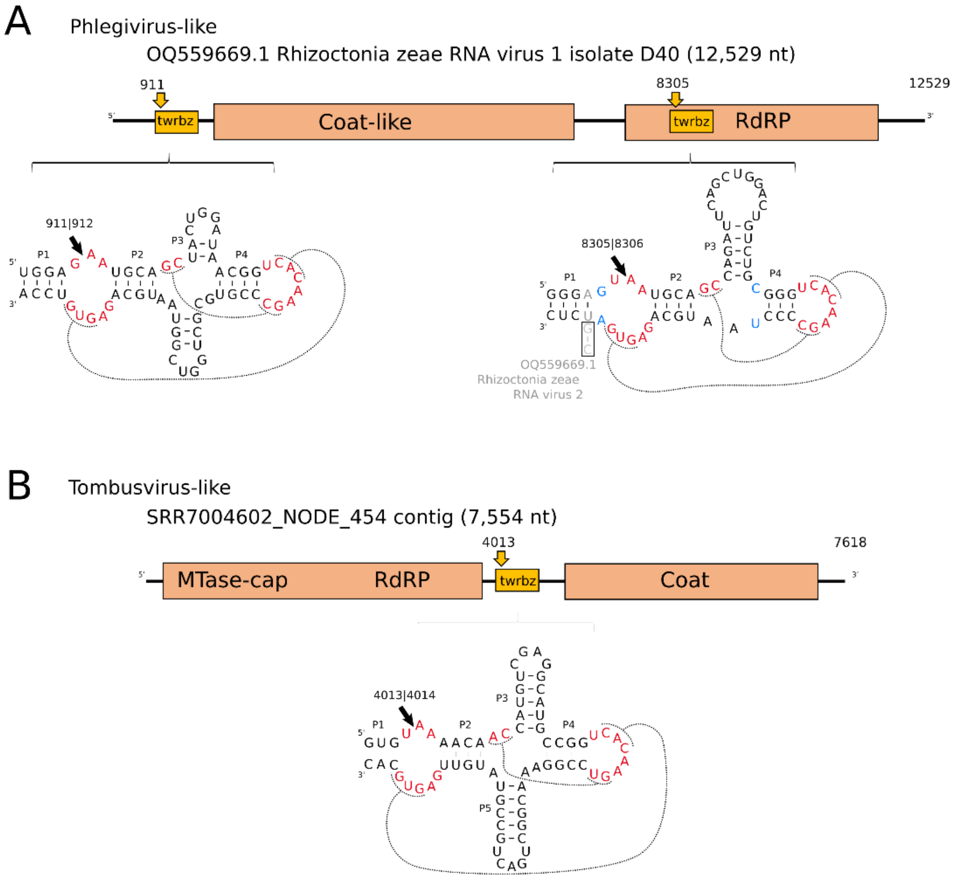

Supplementary Fig. 6. 5’ RACE of Brassica campestris chrysovirus 1 (BcCV-1) from infected cabbage Torzella plants. (A) Schematic representation of the BcCV-1 HHRs in the 5’-UTRs of each of the three genomic RNA segments. (B) Schematic representation of the aproximated locations of the oligos used for the RACE experiments and the expected sizes of the PCR products resulting from the full-length and self-cleaved RNAs (C) Agarose gel analysis of 5’ RACE PCR of BcCV-1 RNA1, RNA2 and RNA3 (lanes 1, 2 and 3, respectively) shows two bands corresponding to the amplification of the full-length (upper and more abundant band) and the self-cleaved (lower band) RNAs. For the RNA1 (lane 1), a nested PCR, using a reverse BcCV-1 RNA1 (BcCV1-RNA1-88NRev, Supplementary Table S2), nested to the primer used in the first RACE amplification (BcCV1-RNA1-87Rev, Supplementary Table S2), is shown. M, DNA molecular weight marker (100 bp DNA ladder, Thermo Fisher). (D) Sequencing electropherograms of the cloned RACE product of smaller size for each RNA genomic segment. The 5’ end coincides with that predicted to be generated by the HHR self-cleavage (indicated by arrows).

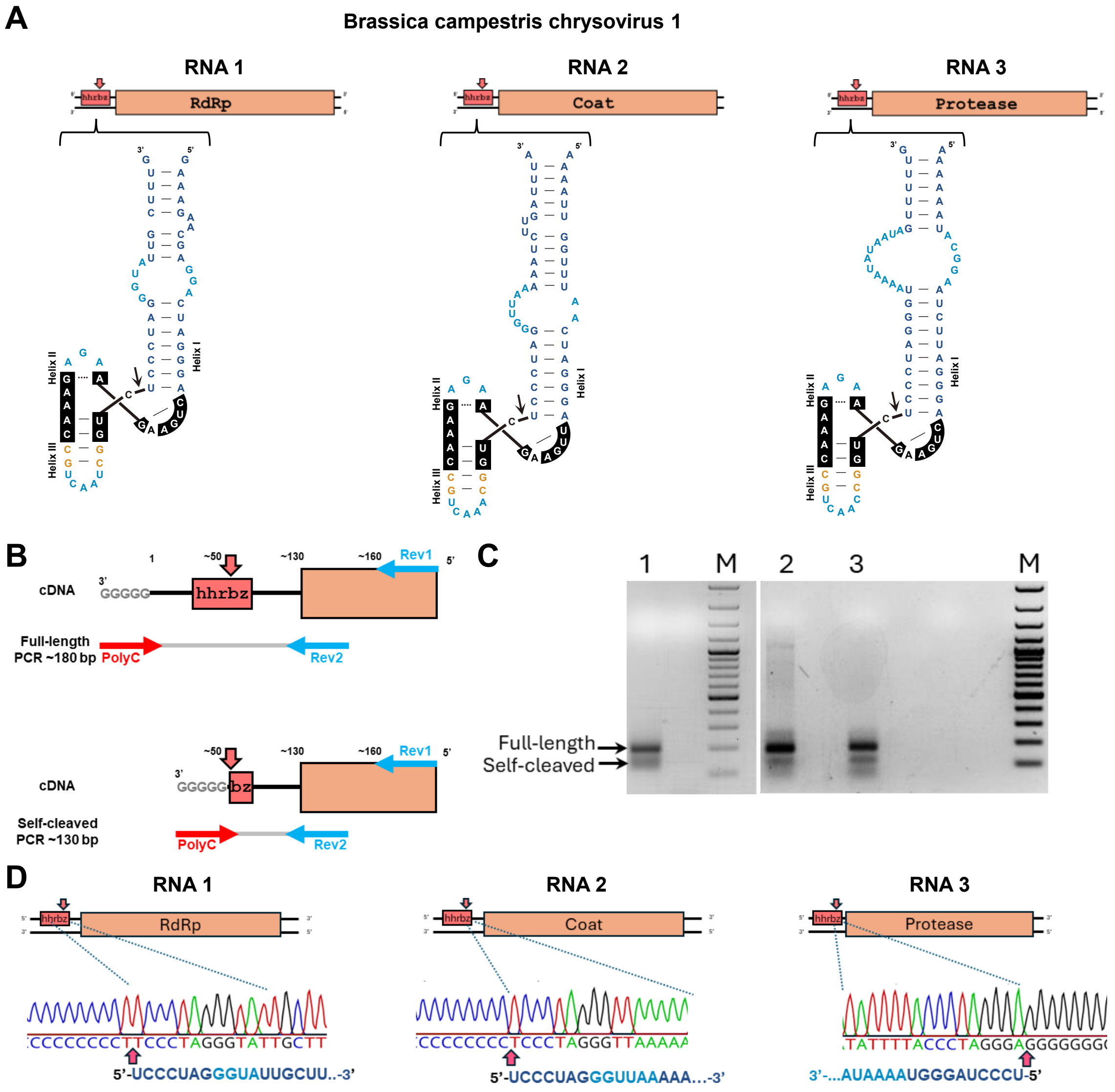

Supplementary Fig. 7. 5’ RACE of Gnomoniopsis castaneae chrysovirus 1 (GcCV-1) from infected *G. castaneae* isolate 1(44). Schematic representation of GcCV-1 RNAs, hammerhead ribozymes (HHR) secondary structure and sequencing electropherograms of the cloned RACE product of smaller size for each (A) RNA 1, (B) RNA 2 (C) RNA 3 and (D) RNA 4 genomic segments. HHRs are present in all the 5’-UTR of RNAs, whereas RNA3 shows a second intergenic HHR motif between the two conserved ORFs (3a and 3b). The 5’ ends of the sequencing electropherograms coincide with those predicted to be generated by the HHR self-cleavage (indicated by arrows). (E) Agarose gel analysis of 5’ RACE PCR (lane 1) and 3’RACE PCR (lane 2) of GcCV-1 RNA2. Lane 1 shows two bands corresponding to the amplification of the full-length (upper band) and the self-cleaved (lower band) RNAs. M; DNA molecular weight marker of 100 bp DNA (Thermo Fisher). RdRp; RNA dependent RNA polymerase. CP; coat protein. ORF; open reading frame.

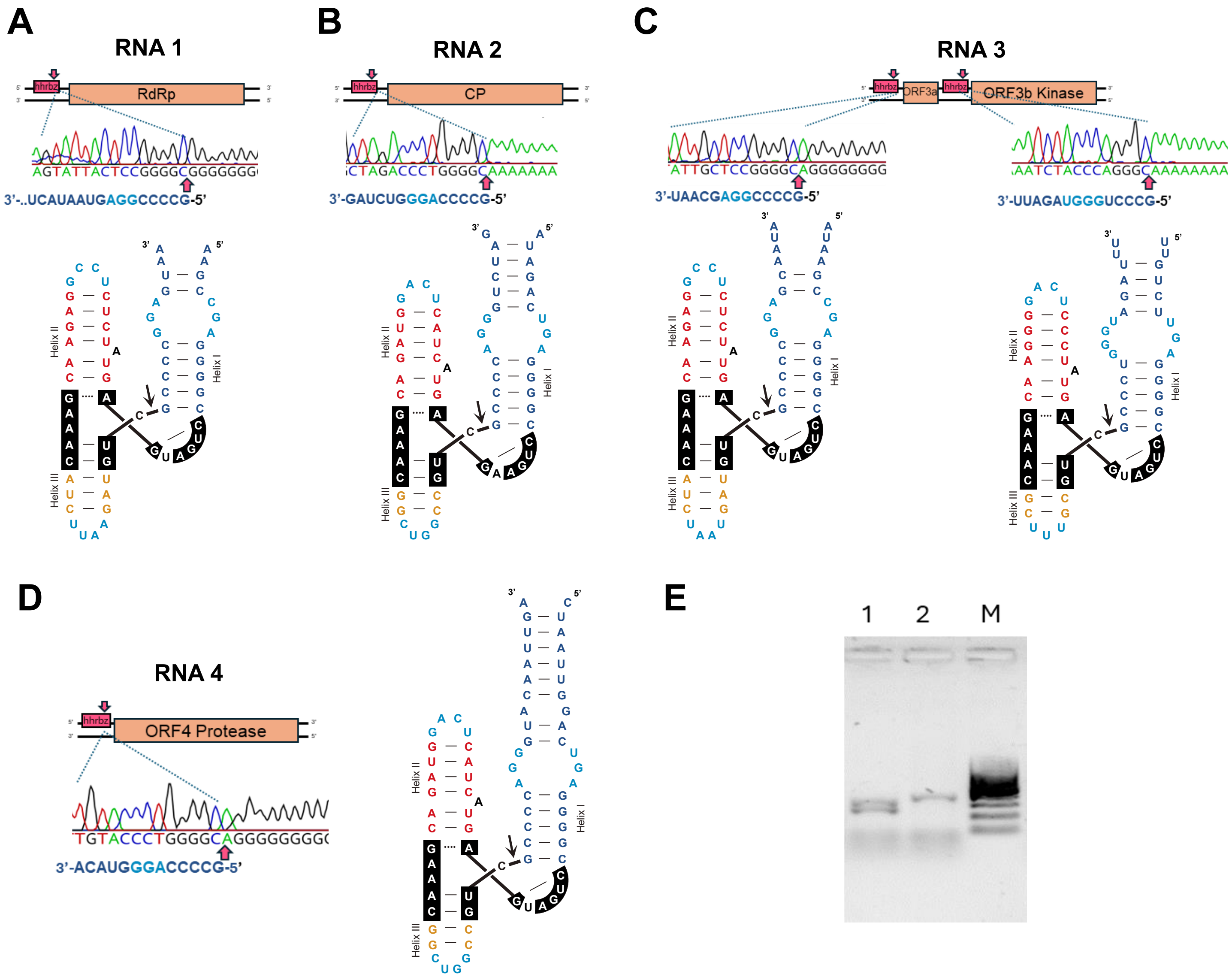

Supplementary Fig. 8. 5’ RACE of Pleospora typhicola fusarivirus 1 (PtFV1) from infected MUT 4379 (62). (A) Genome organization, with the position of the hammerhead ribozyme (HHR) placed in the intergenic region. The secondary structure of the ribozyme is also displayed (right), as well as the sequence electropherogram showing the 5’ end of the cleavage site (red arrow). (B) Northern blot analysis of RNA extracts from virus-infected isolate MUT4379 and virus-free *Rhizoctonia solani* isolate IEE-16(77). On the left panel, red arrows indicate the PtFV1 genomic RNA (gRNA) and PtFV1 sgRNA positions, while green arrows indicate some possible cross-hybridization with the two fungal ribosomal RNAs. The right panel shows a methylene blue stain of the membrane displaying the two ribosomal RNA bands.

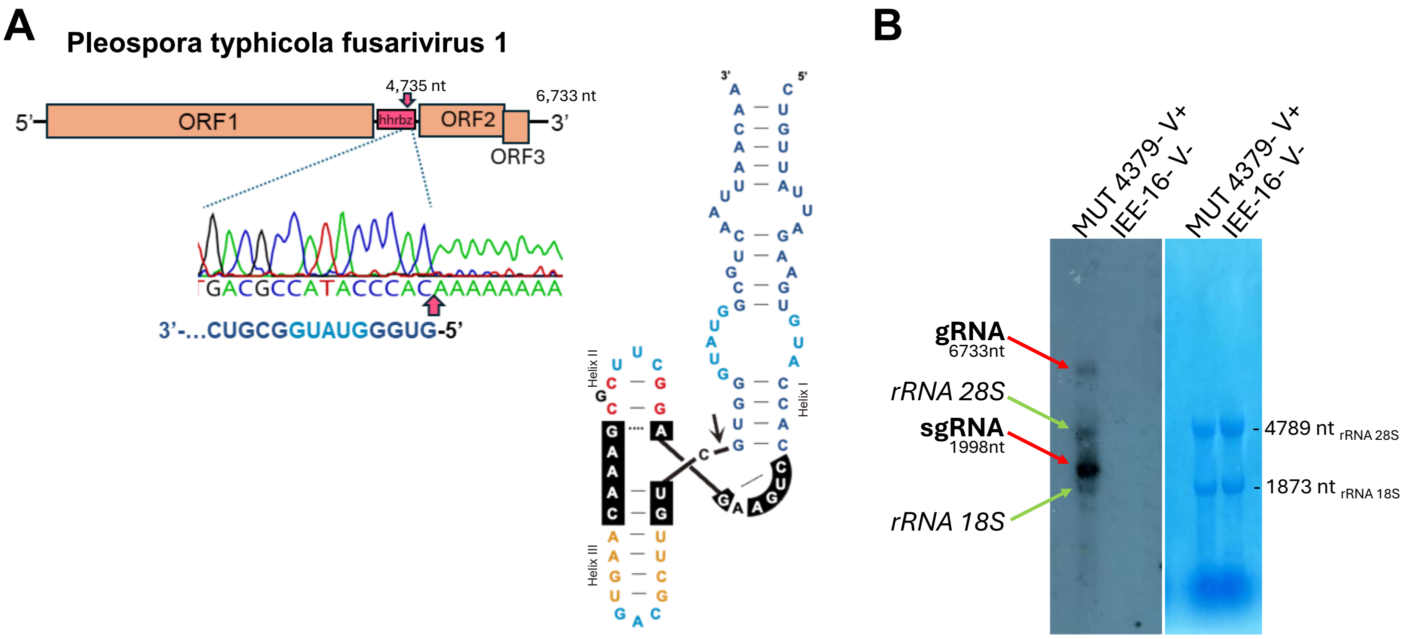

Supplementary Fig. 9. (A) Top: schematic representation of a type I hammerhead ribozyme (HHR) from Schistosoma mansoni with tertiary loop-loop interactions indicated by dotted lines. Bottom: 3D-structure (pdb 3ZD5) showing the central conserved core in black and the three stems I, II, and III in blue, red, and yellow, respectively; loops are not shown. Similarly, schematic (top) and 3D structures (bottom) of (B) the Hv145S-RNA2 HHR (predicted through the Alphafold 3 server(78)), (C) Hepatitis A virus (HAV) IRES domain V (pdb 6mwn)(76) and (D) the J-K domain of the encephalomyocarditis virus (EMCV) IRES element (pdb 2nbx)(74).

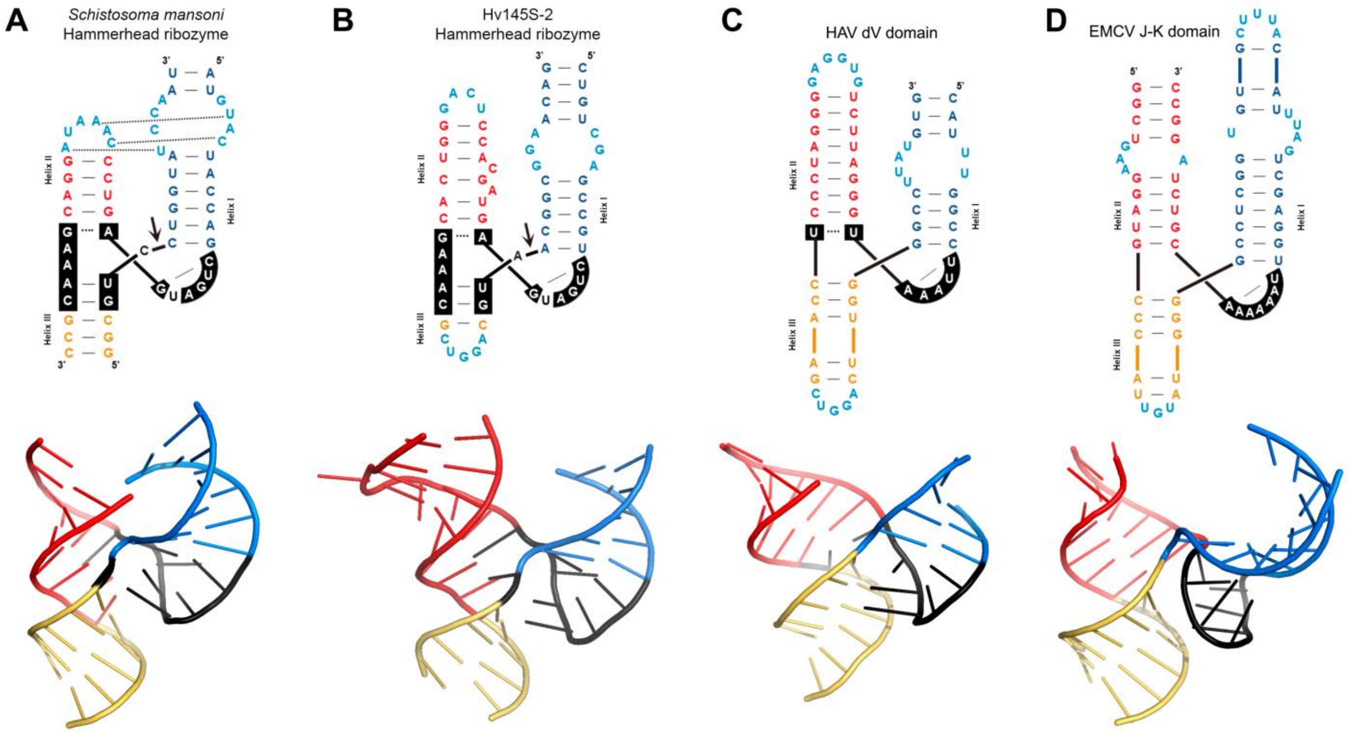

Supplementary Fig. 10. Three dimensional models for (A) the hammerhead ribozyme from *Schistosoma mansoni* retrozymes (pdb 3ZD5, central core and stems, but not interacting loops 1 and 2, are shown), (B) the human Hepatitis Delta ribozyme (pdb 1DRZ), (C) the Hepatitis A virus IRES dV core domain (pdb 6mwn) and (D) the encephalomyocarditis virus IRES J-K core domain (pdb 2nbx).

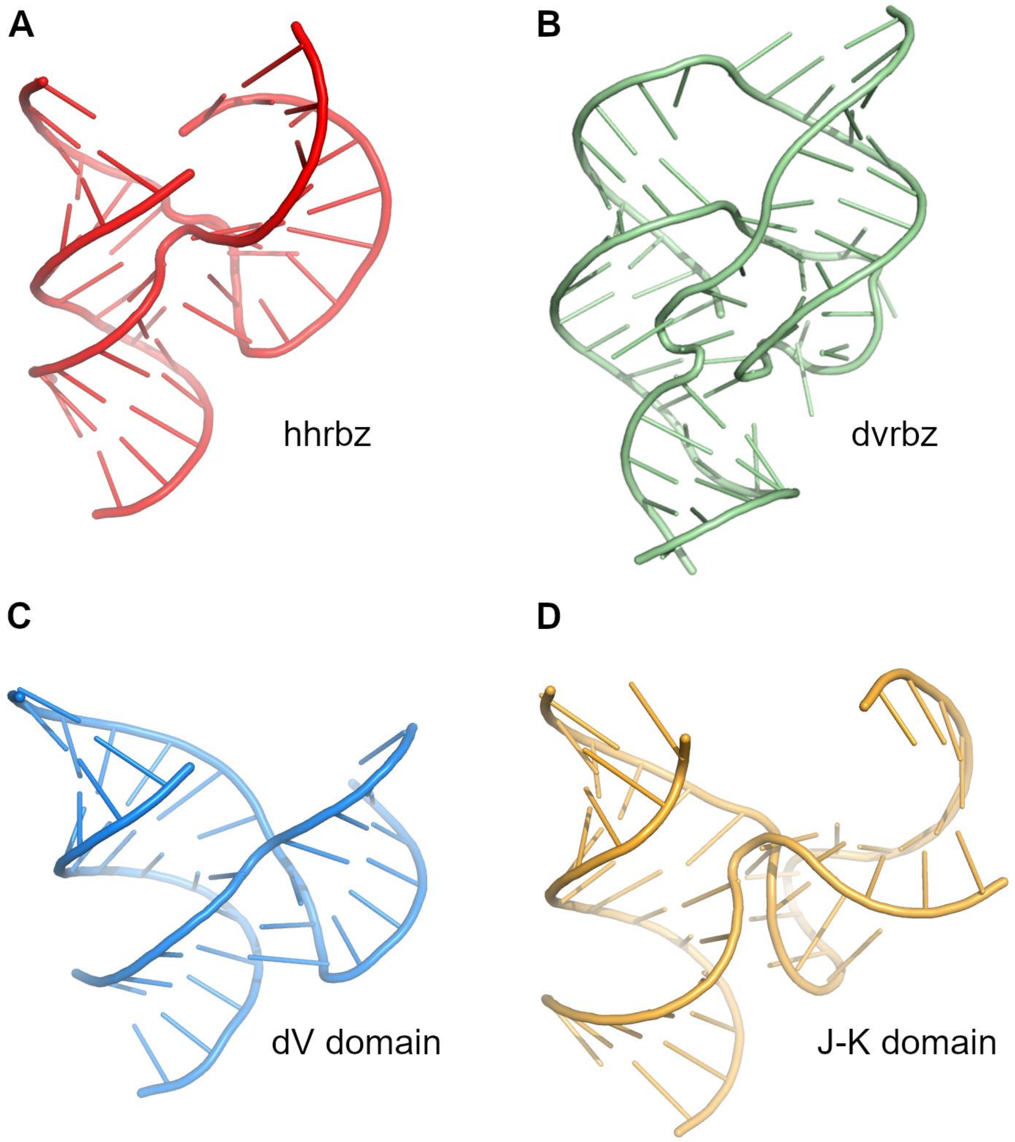

Supplement: Supplementary file 1 [file gkag1106_supplemental_files.zip › supplementary.pdf]
